# Supplementary material for: Experience, circuit dynamics, and forebrain recruitment in larval zebrafish prey capture
Source: eLife. 2020 Sep 28;9:e56619. doi: 10.7554/eLife.56619 (PMC7561350; doi:10.7554/eLife.56619)
Supplement: Supplementary file 1. [file elife-56619-supp1.docx]

**Supplementary file 1.** P-values for permutation test comparison of average fluorescence trace before and after eye convergence in experienced fish for different brain regions (Figure 2H).

**Brain region p-Value**

Telencephalon 0.027

Habenula 0.003

Pretectum (contra) < 0.001

Pretectum (ipsi) 0.697

Tectal neuropil (contra) 0.011

Tectal neuropil (ipsi) 0.018

Tectal PVN < 0.001

Cerebellum < 0.001

Crista cerebellaris 0.001

Hindbrain < 0.001
