# Supplementary material for: Experience, circuit dynamics, and forebrain recruitment in larval zebrafish prey capture
Source: eLife. 2020 Sep 28;9:e56619. doi: 10.7554/eLife.56619 (PMC7561350; doi:10.7554/eLife.56619)
Supplement: Supplementary file 2. — For each link, differences between experienced and naïve fish are assessed. Threshold for significance after FDR-BH correction is p=0.0017 (see Materials and methods). [file elife-56619-supp2.docx]

**Supplementary file 2.** P-values for comparisons of Granger-causality links in the visual areas (matrices presented in Fig 4.). For each link, differences between experienced and naïve fish are assessed. Threshold for significance after FDR-BH correction is p = 0.0017 (see Materials and Methods).

| **Visual Areas - Evoked** | | | | | | |
| --- | --- | --- | --- | --- | --- | --- |
| **Region** | **1** | **2** | **3** | **4** | **5** | **6** |
| **1** |  | 0.38 | 0.21 | 0.48 | 0.18 | 0.35 |
| **2** | 0.30 |  | 0.12 | 0.43 | 0.18 | 0.36 |
| **3** | 0.18 | 0.39 |  | 0.32 | 0.48 | 0.07 |
| **4** | 0.15 | 0.33 | 0.23 |  | 0.30 | 0.36 |
| **5** | 0.11 | 0.29 | 0.30 | 0.26 |  | 0.09 |
| **6** | 0.23 | 0.19 | 0.29 | 0.16 | 0.16 |  |
|  | | | | | | |
| **Visual Areas - Spontaneous** | | | | | | |
| **Region** | **1** | **2** | **3** | **4** | **5** | **6** |
| **1** |  | 0.49 | 0.24 | 0.49 | 0.46 | 0.35 |
| **2** | 0.40 |  | 0.37 | 0.43 | 0.40 | 0.16 |
| **3** | 0.10 | 0.46 |  | 0.34 | 0.43 | 0.29 |
| **4** | 0.09 | 0.09 | 0.36 |  | 0.13 | 0.50 |
| **5** | 0.18 | 0.01 | 0.42 | 0.40 |  | 0.44 |
| **6** | 0.31 | 0.46 | 0.30 | 0.41 | 0.48 |  |
