# Supplementary material for: Experience, circuit dynamics, and forebrain recruitment in larval zebrafish prey capture
Source: eLife. 2020 Sep 28;9:e56619. doi: 10.7554/eLife.56619 (PMC7561350; doi:10.7554/eLife.56619)
Supplement: Supplementary file 3. — P-values for comparisons of directed (causal) interactions between visual and motor areas between experienced and naïve fish (matrices presented in Figure 5A), and strongest and weakest hunters among experienced fish (matrices presented in Figure 5—figure supplement 1A). Threshold for significance after FDR-BH correction is top: p=3.E-04, and bottom p=0.003. Links that are significantly different between groups are highlighted in yellow. [file elife-56619-supp3.docx]

| **Supplementary file 3.** P-values for comparisons of directed (causal) interactions between visual and motor areas between experienced and naïve fish (matrices presented in Fig 5A), and strongest and weakest hunters among experienced fish (matrices presented in Fig 5, figure supplement 1A). Threshold for significance after FDR-BH correction is top: p = 3.E-04, and bottom p = 0.003. Links that are significantly different between groups are highlighted in yellow.  **Visual and motor areas - Experienced and Naïve fish (Fig. 5A), evoked** | | | | | | | |
| --- | --- | --- | --- | --- | --- | --- | --- |
| **Region** | **1** | **2** | **3** | **4** | **5** | **6** | **7** |
| **1** |  | 0.11 | 0.03 | 0.36 | 0.39 | 0.22 | 0.15 |
| **2** | 0.36 |  | 0.10 | 0.18 | 0.33 | 0.18 | 0.35 |
| **3** | 0.16 | 0.21 |  | 0.24 | 0.49 | 0.27 | 0.13 |
| **4** | 0.01 | 0.01 | 0.32 |  | 0.49 | 0.24 | 0.28 |
| **5** | 3.E-04 | 0.07 | 0.02 | 0.21 |  | 0.06 | 0.36 |
| **6** | 0.04 | 0.01 | 0.04 | 0.49 | 0.31 |  | 0.20 |
| **7** | 0.01 | 3.E-03 | 0.02 | 0.40 | 0.11 | 0.19 |  |
|  | | | | | | |  |
| **Visual and motor areas - Strong vs Weak hunters (Fig. 5, fig. suppl. 1A), evoked** | | | | | | | |
| **Region** | **1** | **2** | **3** | **4** | **5** | **6** | **7** |
| **1** |  | 0.21 | 0.17 | 0.48 | 0.33 | 0.09 | 0.17 |
| **2** | 0.22 |  | 0.23 | 0.23 | 0.16 | 0.30 | 0.30 |
| **3** | 0.16 | 0.46 |  | 3.E-12 | 6.E-09 | 3.E-03 | 4.E-04 |
| **4** | 0.07 | 0.16 | 0.49 |  | 0.14 | 0.17 | 0.07 |
| **5** | 0.37 | 0.16 | 0.33 | 0.11 |  | 0.29 | 0.40 |
| **6** | 0.21 | 0.34 | 0.37 | 0.28 | 0.02 |  | 0.07 |
| **7** | 0.43 | 0.34 | 0.47 | 0.35 | 0.31 | 0.17 |  |
